# The cultural dimension of intergroup conspiracy theories

**DOI:** 10.1111/bjop.12471

**Published:** 2020-08-13

**Authors:** Jan‐Willem van Prooijen, Mengdi Song

**Affiliations:** ^1^ Department of Experimental and Applied Psychology VU Amsterdam The Netherlands; ^2^ The Netherlands Institute for the Study of Crime and Law Enforcement (NSCR) Amsterdam The Netherlands

**Keywords:** conspiracy theories, culture, power distance, collective narcissism, outgroup threat

## Abstract

Although conspiracy theories are ubiquitous across times and cultures, research has not investigated how cultural dimensions may predict conspiracy beliefs. The present research examined intergroup conspiracy beliefs in United States and Chinese samples at the peak of the trade war. In two studies (one pre‐registered; total *N* = 1,092), we asked US participants to what extent they believed Chinese institutions and companies were conspiring against the United states and Chinese participants to what extent they believed US institutions and companies were conspiring against China. Results revealed that such beliefs were stronger among Chinese than US participants due to higher power distance values and vertical collectivism. In particular, these cultural dimensions were associated with increased psychological involvement in intergroup conflict (as reflected by higher levels of collective narcissism and perceived outgroup threat), which in turn predicted intergroup conspiracy beliefs. Exploratory analyses suggested that particularly power distance values mediate these effects. We conclude that cultural dimensions that promote hierarchy in society are associated with increased intergroup conspiracy beliefs.

## Background

Conspiracy theories have occurred across times and cultures. Defying the popular notion that we now live in an ‘age of conspiracism’, conspiracy theories were rampant in the entire 20th century, the dark ages, the Roman empire, and ancient Greek mythology (Butter & Knight, 2020; Pagan, 2008; Uscinski & Parent, 2014; Van Prooijen & Douglas, 2017, 2018). Furthermore, conspiracy theories have been observed across cultures worldwide, including various countries in Eastern Europe, Asia, and the Middle East (Gentzkow & Shapiro, 2004; Mashuri & Zaduqisti, 2015; Swami, 2012; Van Prooijen & Van Vugt, 2018). Conspiracy theories are also common in traditional societies – including native tribes in the Amazon and villages in rural parts of Africa – where people often ascribe their group’s misfortune to sorcery committed by a conspiracy in an enemy village (Chagnon, 1988; West & Sanders, 2003). But despite the cultural ubiquity of conspiracy theories, research hitherto has not investigated how culture may be associated with conspiracy beliefs. The present research was designed to fill this void by testing how power distance values and vertical collectivism predict conspiracy beliefs, in samples collected in the United States and China.

A common definition of a conspiracy theory is an explanatory belief that a group of actors colludes in secret to attain some malevolent goal (Douglas, Sutton, & Cichocka, 2017; Van Prooijen, 2018). Conspiracy beliefs can emerge both as a trait and as a state. People structurally differ in their conspiracy mentality, that is, a trait‐like pre‐disposition to ascribe events in the world to the causal actions of hostile conspiracies (Imhoff & Bruder, 2014). But people also differ in their belief in specific conspiracy theories, such as beliefs that climate change is a hoax fabricated by scientists, that the CIA killed JFK, or that the virus causing COVID‐19 was created in the laboratory as a bioweapon. Such specific conspiracy theories by definition imply suspicious intergroup perceptions: A coalition or outgroup perceived as hostile (e.g., a government; a secret service agency; a minority group) allegedly colludes in secret to harm a valued ingroup (e.g., regular citizens), leading scholars to raise the term ‘intergroup conspiracy theories’ (Cichocka, Marchlewska, Golec de Zavala, & Olechowski, 2016). This intergroup dimension distinguishes conspiracy beliefs from paranoia, which is a more self‐focused psychological state (Imhoff & Lamberty, 2018; Van Prooijen & Van Lange, 2014).

Accordingly, recent theoretical models have asserted that intergroup conflict is key to understand conspiracy beliefs. The existential threat model of conspiracy theories articulates that distressing societal events activate epistemic processes to make sense of the event and the negative emotions associated with it. These sense‐making processes only elicit conspiracy beliefs if a despised outgroup is salient, however. In such cases, people may blame the distressing events on the assumed covert actions of the despised outgroup (Van Prooijen, 2020). These arguments are consistent with the Adaptive Conspiracism Hypothesis (Van Prooijen & Van Vugt, 2018), which describes how the mental structures that produce conspiracy thinking could evolve. This evolutionary framework proposes that lethal intergroup conflict was a realistic and recurring danger in the history of humanity, putting significant selection pressure on a tendency to overestimate the likelihood that others are secretly forming hostile coalitions (see also Raihani & Bell, 2018).

Intergroup conflict is commonly associated with two dynamic processes, namely ingroup favouritism and a perception of an outgroup as threatening (Riek, Mania, & Gaertner, 2006; Tajfel & Turner, 1979). Empirical research has associated both of these processes to intergroup conspiracy beliefs. First, one robust predictor of intergroup conspiracy belief is collective narcissism, defined as an exaggerated belief in the greatness of one’s ingroup (Golec de Zavala, Cichocka, Eidelson, & Jayawickreme, 2009). This indicator of ingroup favouritism is associated with intergroup conspiracy beliefs about minority groups (Golec de Zavala & Cichocka, 2012) and other countries (Cichocka *et al*., 2016) and also predicts a progressive increase in conspiracy beliefs about political opponents during an election campaign (Golec de Zavala & Federico, 2018).

Second, information that an outgroup poses a threat to ingroup values promotes intergroup conspiracy theories (Mashuri & Zaduqisti, 2015). This is for instance reflected in a polarized US political landscape, where Democrats versus Republicans hold strong conspiracy beliefs about each other (Uscinski & Parent, 2014). Also, ethnic minority groups often perceive high levels of outgroup threat (e.g., due to experiences of discrimination by a dominant majority group), and accordingly, conspiracy beliefs tend to be relatively high among ethnic minorities (Crocker, Luhtanen, Broadnax, & Blaine, 1999; Van Prooijen, Staman, & Krouwel, 2018). Taken together, these findings suggest that variables commonly associated with intergroup conflict – specifically collective narcissism and perceived outgroup threat – predict intergroup conspiracy beliefs.

### Culture and conspiracy theories

Extrapolating these arguments to the present purposes, it appears likely that particularly cultural variables related to psychological involvement in intergroup conflict are associated with intergroup conspiracy beliefs. In the present research, we therefore focus on cultural dimensions that are commonly associated with intergroup conflict, specifically vertical collectivism and power distance values (Hofstede, 1980). Collectivism refers to the extent to which people construe themselves as interdependent with their social environment. Collectivism can take various forms, however, notably horizontal (i.e., perceiving the self as part of a collective where everyone is equal) and vertical (implying within‐group hierarchy, and a willingness to submit to group authorities). Particularly, vertical collectivism is associated with intergroup competition and hostile intergroup perceptions (e.g., Triandis, 1995; Triandis & Gelfand, 1998).

Power distance values refer to cultural norms about the extent to which unequal power distributions in society are considered acceptable or even desirable (see also Brockner *et al*., 2001). Interpersonal relations in high power distance cultures hence tend to be relatively autocratic. Power distance values have conceptual parallels with authoritarianism, a psychological trait commonly associated with prejudice and hostile intergroup perceptions (Adorno, Frenkel‐Brunswik, Levinson, & Sanford, 1950). Moreover, empirical research reveals that feelings of powerlessness increase people’s acceptance of inequality based on gender, race, or social class (Van der Toorn *et al*., 2015; Study 4).

In the current research, we investigate whether these cultural dimensions, that both imply acceptance of hierarchy in society, predict conspiracy beliefs through the two dynamic processes associated with intergroup conflict. While vertical collectivism includes a sense of obligation within informal social structures (e.g., one’s family or groups of friends) and power distance refers to acceptance of, and obedience to, formal group authorities (e.g., at work, or in society), both cultural dimensions suggest a norm to sacrifice one’s own interests for the benefit of their group or broader community (Singelis, Triandis, Bhawuk, & Gelfand, 1995). Put differently, vertical collectivism and power distance values include norms to prioritize group goals over personal goals, which may be associated with increased ingroup favouritism (as reflected in collective narcissism). Furthermore, such submission to group goals may also involve a relatively strong tendency to reject outsiders, particularly when these outsiders threaten core values or important resources of the ingroup.

The notion that hierarchy within society, and hence a tendency to submit to informal or formal group authorities, predict the dynamic processes underlying intergroup conflict dovetails with empirical research on conspiracy theories. Conspiracy beliefs are associated with feelings of powerlessness (Abalakina‐Paap, Stephan, Craig, & Gregory, 1999; Imhoff & Lamberty, 2020; Van Prooijen, 2017) and with authoritarianism (Imhoff & Bruder, 2014; Swami, 2012). Moreover, experimental manipulations that induce a lack of control among participants increase belief in conspiracy theories (Whitson & Galinsky, 2008; Van Prooijen & Acker, 2015), as do other concepts related to feelings of powerlessness such as threats to the societal status quo (Jolley, Douglas, & Sutton, 2018), and self‐uncertainty (Van Prooijen, 2016). These previous findings, however, did not test the role of hierarchical social relations as a cultural phenomenon nor did they test whether the link between cultural hierarchy and conspiracy beliefs is associated with collective narcissism and perceived outgroup threat.

In the present research, we investigate the cultural dimension of intergroup conspiracy theories by soliciting samples from the United States and China. These two countries were suitable for the present purposes given that (a) they were in mutual conflict (i.e., the US–China trade war) at the time we conducted these studies (April and June 2019), enabling us to assess mutual intergroup conspiracy beliefs in a meaningful manner; and (b) their cultures differ in vertical collectivism and power distance values, with China scoring higher on both dimensions than the United States (Hofstede, 1980). We tested in a serial mediation model whether differences between US versus Chinese samples in intergroup conspiracy beliefs would be associated with cultural dimensions that promote hierarchy in society (vertical collectivism and power distance values) and with variables commonly associated with intergroup conflict (collective narcissism and perceived outgroup threat). More specifically, we test a linear structural model that includes paths from culture (United States versus China) to cultural dimensions (power distance values and vertical collectivism); from cultural dimensions to variables associated with intergroup conflict (collective narcissism and perceived outgroup threat); and from variables associated with intergroup conflict to intergroup conspiracy beliefs.

### Open practices statement

The first study was exploratory; the second study was confirmatory and pre‐registered. All materials, data, analysis scripts, and the pre‐registration of Study 2 are publicly available on the Open Science Framework (https://osf.io/sfy5g/). In the Appendix S1, we disclose all the measures assessed in the questionnaires. Both studies reported here have formal ethical approval (as part of an institutional cluster application by the first author) and were conducted in accordance with the provisions of the declaration of Helsinki.

## STUDY 1

## Method

### Participants

The study was conducted among a total of 493 US and Chinese participants; accordingly, we used an English and a Chinese language version of the questionnaire. US participants were recruited through Amazon’s Mechanical Turk and Chinese participants through Wen‐Juan Wang (a Chinese online platform comparable to Mturk). One participant had a missing value on gender, and 10 participants reported ages that were either unrealistic or severely underage (i.e., ranging from 0 to 12 years), which were coded as missing values. Given that most analyses controlled for age and gender, we dropped these cases, implying a final sample of 482 participants (254 US participants, 228 Chinese participants; 270 men, 212 women; *M*_age_ = 37.08, CI_95%_ [36.10; 38.05]). This sample yields 95% power to detect differences between samples with a small‐to‐medium effect size (*d* = .33) and meets sample size requirements for structural equation modelling of 5–10 participants per estimated parameter (Bentler & Chou, 1987). The study lasted about 10 min, and participants received a small fee for participation.

### Questionnaire

The questionnaire was presented as a study on ‘how people perceive the world’. After giving their informed consent and providing basic demographics, participants responded to a range of measures. All the questions had response scales ranging from 1 (*strongly disagree*) to 7 (*strongly agree*). Besides the measures reported here, we also assessed a range of other, exploratory measures; these are disclosed in the Appendix S1.

We measured *conspiracy mentality* using the 12‐item scale by Imhoff and Bruder (2014), example item ‘There are many very important things happening in the world about which the public is not informed’ (α = .86). Furthermore, we measured *intergroup conspiracy theories* through seven items. In the US sample, we asked to what extent participants believed that Chinese institutions and companies are conspiring against the United States (example items ‘The secret agency of China has been trying to influence political decision‐making in America’, and ‘The Chinese government is secretly conspiring to harm America’). Likewise, in the Chinese sample we asked to what extent participants believed that US companies and institutions are conspiring against China (example items ‘The secret agency of America has been trying to influence political decision‐making in China’, and ‘The American government is secretly conspiring to harm China’). These seven items yielded a reliable scale (α = .94; full set of items in the Appendix S1).

As cultural variables, we measured the four‐item power distance scale by Brockner *et al*. (2001), example item: ‘There should be established ranks in society with everyone occupying their rightful place regardless of whether that place is high or low in ranking’ (α = .85). Moreover, we measured the 8‐item vertical collectivism scale (Singelis *et al*., 1995), example item ‘I would sacrifice an activity that I enjoy very much if my family did not approve of it’ (α = .76).

We measured collective narcissism through a 5‐item scale (Golec de Zavala & Federico, 2018), example item: ‘If the United States/China had a major say in the world, the world would be a much better place’ (α = .89). Finally, we measured perceived outgroup threat with three items, example item: ‘China/the United States poses a threat to the national interests of the United States/China’ (α = .80).

## Results

The means, confidence intervals, and intercorrelations of the measured variables are displayed in Table 1. We first investigate measurement invariance, sample characteristics, and sample effects. After this, we test our line of reasoning through structural equation modelling. Measurement invariance analyses and structural equation modelling were conducted using the *lavaan* package in *R* (Rosseel, 2012). As indicators of acceptable model fit, we considered the CFI (>.90), the RMSEA (<.08) and the SRMR (<.08).

**Table 1:** Means, confidence intervals, and intercorrelations of the measured variables (Study 1)

	Total sample		US sample		China sample		Correlation table					
	*M*	CI_95%_	*M*	CI_95%_	*M*	CI_95%_	1	2	3	4	5	6
1. Conspiracy mentality	4.59	[4.50; 4.67]	4.60	[4.47; 4.74]	4.57	[4.48; 4.66]	–					
2. Intergroup conspiracy theories	4.34	[4.21; 4;46	3.70	[3.54; 3.87]	5.04	[4.90; 5.19]	.44	–				
3. Power distance values	3.85	[3.71; 3.98]	2.92	[2.76; 3.07]	4.88	[4.76; 5.00]	.21	.58	–			
4. Vertical collectivism	4.67	[4.60; 4.75]	4.50	[4.40; 4.61]	4.86	[4.76; 4.96]	.29	.42	.51	–		
5. Collective narcissism	4.27	[4.14; 4.40]	3.56	[3.38; 3.72]	5.06	[4.94; 5.19	.23	.61	.74	.56	–	
6. Perceived outgroup threat	4.53	[4.42; 4.65]	4.30	[4.13; 4.46]	4.80	[4.66; 4.95]	.33	.73	.41	.39	.47	–

Note

Means are on a scale ranging from 1 (lowest) to 7 (highest). All correlations were significant at *p* < .001.

### Measurement invariance analyses

We specified three nested and increasingly restricted models to test for configural invariance, metric invariance, and scalar invariance. Metric and scalar invariance were tested using the thresholds of ΔCFI < −.010; ΔRMSEA < .015; and ΔSRMR < .030 (Chen, 2007). First, we tested a full five‐factor model including all items of the five measured variables (power distance, vertical collectivism, collective narcissism, outgroup threat, and intergroup conspiracy beliefs). The basic configural model (testing the five‐factor structure across cultural samples) had an acceptable fit according to one indicator (SRMR = .080), but a marginal fit according to the two other indicators (CFI = .842; RMSEA = 0.086, CI_90%_[0.081; 0.091), χ^2^(628, *N* = 482) = 1,744.72, *p* < .001. The metric model (restricting factor loadings to be equal across cultural samples) did not deviate from the configural model according to all indicators (ΔCFI = −.008; ΔRMSEA = .001; ΔSRMR = .006; Δχ^2^[22, *N* = 482] = 77.97, *p* < .001), and the scalar model (also restricting intercepts to be equal across cultural samples) did not deviate from the metric model according to two out of three indicators (ΔCFI = −.026; ΔRMSEA = .004; ΔSRMR = .008; Δχ^2^[22, *N* = 482] = 202.64, *p* < .001).

Given the marginal fit of the basic configural model, we explored the data for psychometrically problematic items (Putnick & Bornstein, 2016). It turned out that the last four items of the vertical collectivism scale had low loadings in the US sample (<.50; Hooper, Coughlan, & Mullen, 2008). We therefore again tested the five‐factor model for measurement invariance after dropping these items. The fit of the basic configural model was acceptable (CFI = .892; RMSEA = .080, CI_90%_[0.074; 0.086]; SRMR = .072; χ^2^(440, *N* = 482) = 1,116.10, *p* < .001). Moreover, the metric model did not deviate from the configural model according to all indicators (ΔCFI = −.007; ΔRMSEA = .001; ΔSRMR = .003; Δχ^2^[18, *N* = 482] = 64.15, *p* < .001) and the scalar model did not deviate from the metric model according to two out of three indicators (ΔCFI = −.026; ΔRMSEA = .007; ΔSRMR = .010; Δχ^2^[18, *N* = 482] = 177.68, *p* < .001). Only the CFI was marginal, which might be due to the complexity of the model (i.e., 5 factors in two samples). Specifically, larger models are associated with a decreased fit of the CFI, although the current sample size should offer at least some protection against this (Shi, Lee, & Maydeu‐Olivares, 2019).

In sum, these finding suggest full measurement invariance for all scales except vertical collectivism, which displays partial measurement invariance (indicated if at least half of the items of a scale is invariant across samples; Putnick & Bornstein, 2016). We conclude that our measures have sufficient psychometric properties to warrant statistical comparisons between the US versus Chinese sample.

### Sample characteristics

Independent *t*‐tests revealed that as compared to the US sample, the Chinese sample was younger (*M*_US_ = 39.87 years, CI_95%_[38.24; 41.40]; *M*_China_ = 33.96 years, CI_95%_[32.92; 35.01]), *t*(480) = 6.15, *p* < .001, *d* = .57 and higher educated (*M*_US_ = 2.67, CI_95%_[2.59; 2.76]; *M*_China_ = 3.02, CI_95%_[2.95; 3.08]), *t*(480) = −6.24, *p* < .001, *d* = .58. Moreover, the two samples had different gender distributions (United States: 155 men, 99 women; China: 115 men, 113 women), χ^2^(1, *N* = 482) = 5.46, *p* = .019. We therefore statistically controlled for these demographics in all of our analyses below (results were similar without these statistical control variables).

### Sample effects

We first conducted a 2(sample) × 2(type of conspiracy belief: conspiracy mentality vs. intergroup conspiracy theories) ANOVA with the latter factor included as within‐subjects factor. These results reveal a significant sample x type of conspiracy belief interaction, *F*(1, 477) = 164.22, *p* < .001; η^2^ = .26. The US and Chinese samples did not differ in conspiracy mentality, *F* < 1, but intergroup conspiracy theories were substantially higher in the Chinese sample than in the US sample, *F*(1, 477) = 119.59, *p* < .001; η^2^ = .20 (see Table 1).

Furthermore, we conducted a series of ANOVAs on the remaining variables. These revealed that as compared to the US sample, the Chinese sample scored higher on power distance values, *F*(1, 477) = 373.11, *p* < .001; η^2^ = .40, vertical collectivism, *F*(1, 477) = 20.78, *p* < .001; η^2^ = .04, collective narcissism, *F*(1, 477) = 162.29, *p* < .001; η^2^ = .25, and perceived outgroup threat, *F*(1, 477) = 17.14, *p* < .001; η^2^ = .035 (see Table 1).

### Structural equation modelling

In the model, we specified paths from cultural sample to the cultural variables (power distance values and vertical collectivism), from the cultural variables to the intergroup conflict variables (collective narcissism and perceived outgroup threat), and from the intergroup conflict variables to intergroup conspiracy theories. We controlled for gender, age, and education in each specified path. Given that contextual factors other than culture (e.g., the trade war) may have contributed to the observed difference between the Chinese versus US samples in intergroup conspiracy beliefs, we also included a direct link between sample and intergroup conspiracy beliefs. Finally, we allowed the two cultural variables, and the two variables associated with intergroup conflict, to covary.

**Figure 1:**
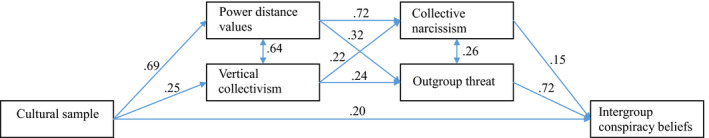
Structural equation model (Study 1; completely standardized solution). Samples were United States (0) and China (1). All paths included gender, age, and education as control variables. All regression coefficients are significant (*p*s < .001). CFI = .901; RMSEA = .068; SRMR = .059; χ^2^(377, *N* = 482) = 1,216.27, *p* < .001. [Colour figure can be viewed at wileyonlinelibrary.com]

The model displayed an acceptable fit according to two indicators (RMSEA = .070, CI_90%_[0.066; 0.074]; SRMR = .060) and a marginal fit according to the third indicator (CFI = .889), χ^2^(460, *N* = 482) = 1,370.60, *p* < .001. To improve model fit, we then inspected for items with low factor loadings (<.50) on the latent variables, as these commonly are considered error (Hooper *et al*., 2008). This led us to remove one item (last item of the vertical collectivism scale, ‘Children should feel honoured if their parents receive a distinguished award’, estimate = 0.316). The final model had an acceptable fit (CFI = .901; RMSEA = .068, CI_90%_[0.066; 0.072]; SRMR = 0.059; χ^2^[377, *N* = 482] = 1,216.27, *p* < .001), and is displayed in Figure 1.

All the individual regression coefficients in the model were significant (*p*s < .001). Furthermore, all four serial indirect effects (from cultural sample to intergroup conspiracy beliefs) were significant: For the path through power distance and collective narcissism, *B* = 0.190, *SE* = .056, CI_95%_[0.081; 0.300], *z* = 3.405, *p* = .001; for the path through power distance and outgroup threat, *B* = 0.402, *SE* = .093, CI_95%_[0.220; 0.584], *z* = 4.333, *p* < .001; for the path through vertical collectivism and collective narcissism, *B* = 0.021, *SE* = .009, CI_95%_[0.004; 0.039], *z* = 2.409, *p* = .016; for the path through vertical collectivism and outgroup threat, *B* = 0.112, *SE* = .040, CI_95%_[0.033; 0.191], *z* = 2.778, *p* = .005. Together, these finding provided strong support for the model.

In the light of the psychometric problems of the vertical collectivism scale (see also measurement invariance analyses), we also tested a more parsimonious model where we dropped this variable from the model. This parsimonious model had an acceptable fit (CFI = .920; RMSEA = .077, CI_90%_[0.071; 0.083]; SRMR = .051; χ^2^[209, *N* = 482] = 804.52, *p* < .001), and the coefficients of all individual paths were significant (*p*s < .001). Furthermore, both serial indirect effects were significant: For the path through power distance and collective narcissism, *B* = 0.229, *SE* = .064, CI_95%_[0.103; 0.355], *z* = 3.570, *p* < .001; for the path through power distance and outgroup threat, *B* = 0.577, *SE* = .079, CI_95%_[0.422; 0.732], *z* = 7.289, *p* < .001. This model is displayed graphically in the supplemental materials (Figure S1). These findings suggest that power distance may be the key cultural dimension underlying intergroup conspiracy beliefs.

## Discussion

The Study 1 results suggest that due to higher power distance values and vertical collectivism levels, Chinese participants reported stronger collective narcissism and outgroup threat than US participants. These intergroup variables, in turn, predicted intergroup conspiracy beliefs. These findings provide preliminary evidence that cultural dimensions related to accepting hierarchy in society (power distance values and vertical collectivism) are associated with increased intergroup conspiracy beliefs.

## STUDY 2

Informed by our theoretical framework and by the Study 1 findings, we designed Study 2 as a direct, confirmatory replication. We pre‐registered the prediction to replicate the model displayed in Figure 1 and formulated the following hypotheses: First, intergroup conspiracy beliefs will be higher in the Chinese than in the US sample (Hypothesis 1). Second, this effect is mediated by the two cultural variables power distance values and vertical collectivism and by the intergroup variables perceived outgroup threat and collective narcissism (Hypothesis 2). We specifically expected that power distance and vertical collectivism would be higher in the Chinese than in the US sample (Hypothesis 2a); that power distance is positively related to perceived outgroup threat and collective narcissism (Hypothesis 2b); that vertical collectivism is positively related to perceived outgroup threat and collective narcissism (Hypothesis 2c); and that perceived outgroup threat and collective narcissism are positively related to intergroup conspiracy beliefs (Hypothesis 2d).

## Method

### Participants

Participants were recruited in the same manner as Study 1. Following our pre‐registered data collection plan, we aimed to collect 300 participants per country (although we expected between 300 and 350 participants in China given that the Wen‐Juan Wang forum offers less control over data collection); accordingly, at the end of data collection we had a total sample of 613 participants. Three participants (two United States, one China) had missing values on gender, leaving a final sample of 610 participants for the analyses (298 US participants, 312 Chinese participants; 330 men, 280 women; *M*_age_ = 34.95, CI_95%_[34.18; 35.73]). This sample yields 95% power to detect differences between samples with a small‐to‐medium effect size (*d* = .27) and again meets sample size requirements for structural equation modelling given the number of estimated parameters in our model (Bentler & Chou, 1987).

### Questionnaire

The questionnaire was the same as in Study 1. We incorporated the same measures of conspiracy mentality (α = .85), intergroup conspiracy beliefs (α = .95), power distance values (α = .84), vertical collectivism (α = .79), collective narcissism (α = .90), and perceived outgroup threat (α = .77).

## Results

The means, confidence intervals, and intercorrelations of the measured variables are displayed in Table 2. We again first analysed measurement invariance, sample characteristics, and sample effects. After this, we tested the hypotheses through structural equation modelling.

**Table 2:** Means, standard deviations, and intercorrelations of the measured variables (Study 2)

	Total sample		US sample		China sample		Correlation table					
	*M*	CI_95%_	*M*	CI_95%_	*M*	CI_95%_	1	2	3	4	5	6
1. Conspiracy mentality	4.62	[4.55; 4.70]	4.59	[4.47; 4.72]	4.65	[4.56; 4.74]	–					
2. Intergroup conspiracy theories	4.52	[4.40; 4.64]	3.79	[3.61; 3.98]	5.21	[5.09; 5.32]	.38	–				
3. Power distance values	3.95	[3.83; 4.07]	3.16	[2.98; 3.34]	4.70	[4.60; 4.81]	.26	.63	–			
4. Vertical collectivism	4.69	[4.61; 4.76]	4.40	[4.27; 4.52]	4.96	[4.88; 5.05]	.19	.47	.60	–		
5. Collective narcissism	4.39	[4.27; 4.51]	3.65	[3.46; 3.85]	5.09	[4.99; 5.19]	.24	.74	.73	.58	–	
6. Perceived outgroup threat	4.50	[4.39; 4.61]	4.34	[4.17; 4.52]	4.65	[4.52; 4.78]	.31	.63	.44	.39	.52	–

Note

Means are on a scale ranging from 1 (lowest) to 7 (highest). All correlations were significant at *p* < .001.

### Measurement invariance analyses

The basic configural five‐factor model had an acceptable fit according to the SRMR and a marginal fit according to the CFI and RMSEA (CFI = .846; RMSEA = .086, CI_90%_[0.082; 0.090]; SRMR = .080), χ^2^(628, *N* = 610) = 2,037.64, *p* < .001. The metric model did not deviate from the configural model according to all indicators (ΔCFI = −.005; ΔRMSEA = .000; ΔSRMR = .007; Δχ^2^[22, *N* = 610] = 85.26, *p* < .001) and the scalar model did not deviate from the metric model according to two out of three indicators (ΔCFI = −.020; ΔRMSEA = .004; ΔSRMR = .004; Δχ^2^[22, *N* = 610] = 207.17, *p* < .001).

We then tested the five‐factor model for measurement invariance after dropping the same four vertical collectivism items as in Study 1. This improved the fit of the basic configural model (CFI = .894; RMSEA = .084, CI_90%_[0.079; 0.089]; SRMR = .069; χ^2^(440, *N* = 610) = 1,394.99, *p* < .001). According to two out of three indicators, the metric model did not deviate from the configural model (ΔCFI = −.016; ΔRMSEA = .001; ΔSRMR = .008; Δχ^2^[18, *N* = 610] = 82.35, *p* < .001), and the scalar model did not deviate from the metric model (ΔCFI = −.018; ΔRMSEA = .004; ΔSRMR = .007; Δχ^2^[18, *N* = 610] = 166.60, *p* < .001). We conclude that our measures are sufficiently invariant to warrant statistical comparisons across cultural samples.

### Sample characteristics

Results again revealed that the Chinese sample was younger (*M*_US_ = 37.34 years, CI_95%_[36.15; 38.53]; *M*_China_ = 32.68 years, CI_95%_[31.75; 33.61]), *t*(608) = 6.11, *p* < .001, *d* = .49 and higher educated (*M*_US_ = 2.82, CI_95%_[2.73; 2.90]; *M*_China_ = 2.99, CI_95%_[2.95; 3.04], *t*(608) = −3.70, *p* < .001, *d* = .28, than the US sample. Moreover, the two samples again had different gender distributions (United States: 178 men, 120 women; China: 152 men, 160 women), χ^2^(1, *N* = 610) = 7.45, *p* = .006. In keeping with our pre‐registered *R*‐script for the confirmatory structural equation models, we statistically controlled for these demographics in all our analyses below (we also ran all analyses without these statistical controls, and results were similar).

### Sample effects

The means per sample are displayed in Table 2. A 2(sample) × 2(type of conspiracy belief: conspiracy mentality vs. intergroup conspiracy theories) ANOVA with the latter factor included as within‐subjects factor indicated a significant sample × type of conspiracy belief interaction, *F*(1, 605) = 147.17, *p* < .001; η^2^ = .20. Consistent with the Study 1 findings, the US and Chinese samples did not differ in conspiracy mentality, *F* < 1, but intergroup conspiracy theories were higher in the Chinese than the US sample, *F*(1, 605) = 255.66, *p* < .001; η^2^ = .20. This latter finding supported Hypothesis 1.

Furthermore, as compared to the US sample, the Chinese sample scored higher on power distance values, *F*(1, 605) = 179.99, *p* < .001; η^2^ = .23, and vertical collectivism, *F*(1, 605) = 46.19, *p* < .001; η^2^ = .07, supporting Hypothesis 2a. On the remaining variables, we again found that the Chinese sample scored higher than the US sample on collective narcissism, *F*(1, 605) = 150.09, *p* < .001; η^2^ = .20, and perceived outgroup threat, *F*(1, 605) = 6.19, *p* = .013; η^2^ = .010.

### Confirmatory analyses

**Figure 2:**
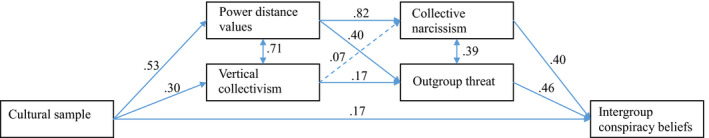
Structural equation model (Study 2; completely standardized solution). Samples were United States (0) and China (1). All paths included gender, age, and education as control variables. Dashed line (vertical collectivism to collective narcissism) is not significant (*p* = .198); vertical collectivism to outgroup threat is significant at*p* = .029; all other regression coefficients are significant at *p* < .001. CFI = .894; RMSEA = .071; SRMR = .056; χ^2^(377; *N* = 610) = 1,540.49, *p* < .001. [Colour figure can be viewed at wileyonlinelibrary.com]

We then tested the main hypotheses, and the overall structural equation model, using the *lavaan* package in *R*. We pre‐registered the CFI and RMSEA as indicators of model fit; although in keeping with Study 1, we also considered the SRMR. Furthermore, we pre‐registered the Study 1 decision to remove items with loadings <.50 to improve model fit (Hooper *et al*., 2008). A first run of the model (with all parameters included) revealed an acceptable fit according to two indicators (RMSEA = .072, CI_90%_[0.068; 0.075]; SRMR = .056) and a marginal fit according to the third indicator (CFI = .887), χ^2^(406; *N* = 610) = 1,672.76, *p* < .001. As in Study 1, we removed the last item of the vertical collectivism scale given a low loading on the latent variable (estimate = 0.323). The results revealed an acceptable fit (with one indicator being marginal and two acceptable): CFI = .894; RMSEA = .071, CI_90%_[0.067; 0.075]; SRMR = .056; χ^2^(377; *N* = 610) = 1,540.49, *p* < .001. Figure 2 displays all the individual paths of this final model.

We then again examined the four serial indirect effects from cultural sample to intergroup conspiracy theories. Results revealed that three out of four indirect effects were significant: For the path through power distance and collective narcissism, *B* = 0.463, *SE* = .064, CI_95%_[0.337; 0.590], *z* = 7.189, *p *< .001; for the path through power distance and outgroup threat, *B* = 0.260, *SE* = .061, CI_95%_[0.140; 0.380], *z* = 4.239, *p* < .001; for the path through vertical collectivism and outgroup threat, *B* = 0.065, *SE* = .032, CI_95%_[0.003; 0.127], *z* = 2.052, *p* = .040. These findings support our hypotheses, except for the serial indirect effect through vertical collectivism and collective narcissism, which was not significant, *B* = 0.024, *SE* = .019, CI_95%_[−0.013; 0.061], *z* = 1.258, *p* = .209.

### Exploratory analyses

In line with Study 1, we again tested a more parsimonious model in which we dropped vertical collectivism and included only power distance values as cultural mediator. The model with path coefficients is displayed in the supplementary materials (Figure S2). This parsimonious model had an acceptable fit (CFI = .909; RMSEA = .082, CI_90%_[0.077; 0.087]; SRMR = .047; χ^2^[209; *N* = 610] = 1,062.19, *p* < .001), and all individual regression coefficients were significant (*p*s < .001). Moreover, both serial indirect effects (from cultural sample to intergroup conspiracy beliefs) were significant: For the path through power distance and collective narcissism, *B* = 0.500, *SE* = .063, CI_95%_[0.377; 0.623], *z* = 7.975, *p* < .001; and for the path through power distance and outgroup threat, *B* = 0.348, *SE* = .050, CI_95%_[0.249; 0.446], *z* = 6.938, *p* < .001.

## Discussion

Study 2 largely replicated the Study 1 findings in a confirmatory and pre‐registered design. Moreover, consistent with Study 1, an exploratory analysis of a more parsimonious model suggests that power distance is the key cultural dimension to predict intergroup conspiracy theories. This prominent role of power distance values is consistent with other findings relating conspiracy beliefs to feelings of powerlessness (e.g., Abalakina‐Paap *et al*., 1999; Jolley *et al*., 2018; Van Prooijen, 2017) and authoritarianism (Imhoff & Bruder, 2014; Swami, 2012), yet expands these previous findings to a cultural context.

## GENERAL DISCUSSION

The current studies were designed to examine the cultural dimension of intergroup conspiracy theories. Results revealed that intergroup conspiracy theories were higher in Chinese samples than in US samples, which was mediated by the cultural dimensions power distance values and vertical collectivism. Consistent with theoretical models asserting that conspiracy beliefs are rooted in perceived intergroup conflict (Van Prooijen, 2020; Van Prooijen & Van Vugt, 2018), the effects of these cultural dimensions were mediated by variables commonly associated with psychological involvement in intergroup conflict (i.e., collective narcissism and perceived outgroup threat). Finally, exploratory analyses suggested that particularly power distance is the key cultural dimension that is associated with intergroup conspiracy theories.

The present research offers three more specific contributions. First, while conspiracy theories occur across times and cultures (e.g., Butter & Knight, 2020; Van Prooijen & Van Vugt, 2018), no research has yet explicitly investigated the role of cultural dimensions in conspiracy theories. The current studies therefore are a preliminary step towards understanding how culture is associated with intergroup conspiracy beliefs. Particularly, cultural dimensions that involve the acceptance of hierarchy in society appear to matter for these beliefs, which may provide a theoretical framework to further examine how conspiracy theories may manifest themselves across cultures. Second, while previous research illuminated that feelings of powerlessness increase conspiracy beliefs (Abalakina‐Paap *et al*., 1999; Van Prooijen, 2017; Van Prooijen & Acker, 2015; Whitson & Galinsky, 2008), the role of power distance values as a cultural dimension was hitherto untested. Combined with earlier research, the present findings underscore that the psychological dynamics associated with formal power relations are important in the psychology of conspiracy theories. Third, while previous research has established empirical links between variables associated with intergroup conflict – notably collective narcissism and perceived outgroup threat – and conspiracy beliefs (e.g., Cichocka *et al*., 2016; Golec de Zavala & Federico, 2018; Mashuri & Zaduqisti, 2015), these associations had not yet been linked to cultural dimensions. The present research provides first evidence that collective narcissism and perceived outgroup threat mediate the relationships between the cultural dimensions under investigation here and intergroup conspiracy beliefs.

The results of the linear structural models are consistent with the notion that acceptance of hierarchy in society implies a norm to submit to group authorities and, hence, to prioritize group goals over personal goals (e.g., Singelis *et al*., 1995; Triandis, 1995). In theorizing on conspiracy theories, however, it is often assumed that feelings of powerlessness are more generally associated with negative reactions towards power, both between and within groups (e.g., Abalakina‐Paap *et al*., 1999; Imhoff & Lamberty, 2020; Van Prooijen, 2017). While this line of reasoning has merit, it is unclear whether it can explain the cultural differences observed here. After all, although power distance values and vertical collectivism were significantly correlated with conspiracy mentality in both studies, there were no differences in conspiracy mentality between the US versus Chinese cultural samples. In the light of the mediating roles of collective narcissism and outgroup threat in our models, the findings appear more consistent with the implications of hierarchical social relations for perceived intergroup conflict to account for the role of culture in conspiracy beliefs.

Related to this point, while the cultural effects observed here did not emerge for conspiracy mentality, they did emerge for intergroup conspiracy beliefs. These findings may imply that cultural dimensions are related to people’s appraisals of specific societal circumstances that involve an antagonistic outgroup as evidence for intergroup conspiracy theories, but also, that cultural dimensions are unrelated to people’s structural pre‐dispositions to perceive hostile conspiracies in the world. Consistent with these findings, authoritarianism – a personality trait involving acceptance of hierarchy – is empirically associated with belief in specific conspiracy theories, but not with generalized conspiracy mentality (Abalakina‐Paap *et al*., 1999; Imhoff & Bruder, 2014; Swami, 2012).

### Strengths, limitations, and future research

The studies presented here have a number of strengths and limitations. Both studies were well‐powered and yielded consistent results. Moreover, one of the studies was pre‐registered before implementation. These considerations suggest that the findings observed here are robust and provide a solid empirical basis for further research on the role of culture in conspiracy beliefs. Moreover, the research took place in a unique setting of samples drawn from two culturally different countries that were involved in mutual conflict at the time when we conducted these studies (i.e., the 2019 US–China trade war). As such, this setting allowed for a meaningful test of the presumed relationships between cultural dimensions, variables associated with intergroup conflict, and intergroup conspiracy theories.

A limitation, however, is that the findings observed here are cross‐sectional, and our model therefore hinges on an assumed sequence of the variables under investigation here. More rigorous designs would be necessary to persuasively validate all the paths in the model. These designs may include experiments, which are superior to mediational analyses in establishing a causal chain (Spencer, Zanna, & Fong, 2005). Also, longitudinal designs may be helpful to establish how cultural dimensions predict fluctuations in intergroup conspiracy beliefs as conflict between groups increases or decreases over time. A second limitation is that it is impossible to ascertain how representative these samples are for the US and Chinese populations, and what factors other than the cultural dimensions measured here may have contributed to the observed differences between our US and Chinese samples (e.g., level of democracy, freedom of press, and so on). We therefore restrict our conclusions to the role of underlying cultural dimensions (i.e., power distance values and vertical collectivism) in intergroup conspiracy theories, without drawing definitive conclusions about the likelihood of Chinese versus US citizens believing specific conspiracy theories, or about the role of other aspects of US versus Chinese society that we did not address in this study.

The measure of intergroup conspiracy beliefs necessarily implied different wordings across cultural samples, with US participants being asked whether Chinese institutions conspire against the United States and Chinese participants being asked whether US institutions conspire against China. One potential problem with this approach is that it may reflect actual, real‐world differences (i.e., maybe the United States actually is conspiring more against China than vice versa). Such an interpretation can be reconciled with only part of the model, however. Specifically, such an interpretation could be consistent with the link between intergroup conspiracy belief and outgroup threat (i.e., possibly the United States is in fact more threatening towards China than vice versa). It is more difficult to see, however, how this interpretation would explain the link between intergroup conspiracy theories and collective narcissism or the cultural variables assessed here. Moreover, the intergroup conspiracy beliefs measure showed no psychometric problems across cultural samples (see measurement invariance analyses). Nevertheless, future research may be designed to more directly exclude this possibility.

While the studies reported here were conducted in 2019, the 2020 global COVID‐19 pandemic underscores the need to further investigate how culture shapes intergroup conspiracy thinking. According to a PEW research poll conducted in mid‐March 2020, 29% of the US population believes that the coronavirus was created in a Chinese laboratory. Around the same time, a Chinese diplomat articulated the conspiracy theory that the US military have brought the corona virus to Wuhan.^1^ These societal developments further illuminate how different cultural groups can use conspiracy theories to blame each other of unfolding crises, which may stimulate or perpetuate international conflict. Understanding the complex cultural processes that play a role in these issues is necessary to develop evidence‐based interventions focused on increasing mutual trust and cooperation.

### Concluding remarks

Conspiracy theories are ubiquitous across times and cultures: They have been widespread across Western and Eastern cultures, modern and traditional societies, ancient and recent civilizations, and so on (Butter & Knight, 2020; Pagan, 2008; West & Sanders, 2003). Such cultural ubiquity has contributed to the theoretical perspective that conspiracy beliefs are rooted in a set of evolved psychological mechanisms (Raihani & Bell, 2018; Van Prooijen & Van Vugt, 2018). But besides such similarities between cultures, it is highly likely that also cultural differences exist in people’s propensity towards conspiracy thinking. The present research is the first to highlight what role cultural dimensions play in people’s susceptibility to intergroup conspiracy theories. The results support a model indicating that cultural dimensions associated with acceptance of hierarchy predict intergroup conspiracy beliefs, due to increased psychological involvement in intergroup conflict. These findings suggest that conspiracy beliefs cannot be understood separately from the cultural context in which they transpire.

## Conflicts of interest

All authors declare no conflict of interest.

## Author contribution

Jan‐Willem van Prooijen (Conceptualization; Formal analysis; Investigation; Methodology; Resources; Supervision; Validation; Visualization; Writing – original draft; Writing – review & editing) Mengdi Song (Conceptualization; Formal analysis; Investigation; Methodology; Project administration; Validation; Writing – review & editing).

## Supporting information


**Appendix S1.** Online supplemental materials.
**Figure S1.** Parsimonious structural equation model in Study 1 (completely standardized solution).
**Figure S2.** Parsimonious structural equation model in Study 2 (completely standardized solution).

## Data Availability

All materials, data, analysis scripts, and the pre‐registration of Study 2 are publicly available on the Open Science Framework (https://osf.io/sfy5g/).

## Footnotes

^1^ For more information on the PEW research poll, see https://www.pewresearch.org/fact‐tank/2020/04/08/nearly‐three‐in‐ten‐americans‐believe‐covid‐19‐was‐made‐in‐a‐lab/; for news about the Chinese diplomat accusing the US military, see https://edition.cnn.com/2020/03/13/asia/china‐coronavirus‐us‐lijian‐zhao‐intl‐hnk/index.html
