# Supplementary material for: The cultural dimension of intergroup conspiracy theories
Source: Br J Psychol. 2020 Aug 13;112(2):455–73. doi: 10.1111/bjop.12471 (PMC8246844; doi:10.1111/bjop.12471)
Supplement: Supplementary file 1 — Appendix S1. Online supplemental materials. Figure S1. Parsimonious structural equation model in Study 1 (completely standardized solution). Figure S2. Parsimonious structural equation model in Study 2 (completely standardized solution). [file BJOP-112-455-s001.docx]

Online Supplemental Materials

for

**The Cultural Dimension of Intergroup Conspiracy Theories**

Below, we will report all the measures included in the questionnaire, both in English (as presented to US participants) and Chinese (as presented to Chinese participants). We first display the measures reported in the manuscript, after which we disclose the measures that were not analyzed in the manuscript. All questions were answered on a scale ranging from 1 (strongly disagree) to 7 (strongly agree).

At the end of the document we also display two supplementary figures (S1 and S2).

**Measures analyzed in the Manuscript:**

**Conspiracy Mentality (Imhoff & Bruder, 2014)**

***English Version***

There are many very important things happening in the world about which the public is not informed.

Those at the top do whatever they want.

A few powerful groups of people determine the destiny of millions.

There are secret organizations that have great influence on political decisions.

I think that the various conspiracy theories circulating in the media are absolute nonsense. (R)

Politicians and other leaders are nothing but the string puppets of powers operating in the background.

Most people do not recognize to what extent our life is determined by conspiracies that are concocted in secret.

There is no good reason to distrust governments, intelligence agencies, or the media. (R)

International intelligence agencies have their hands in our everyday life to a much larger degree than people assume.

Secret organizations can manipulate people psychologically so that they do not notice how their life is being controlled by others.

There are certain political circles with secret agendas that are very influential.

Most people do not see how much our lives are determined by plots hatched in secret.

***Chinese Version***

世界上有许多重要的事情发生，但是公众对此并不知情。

那些在上层的人可以做他们想做的任何事情。

几个拥有权力的群体就可以决定数百万人的命运。

有一些秘密组织，他们对政治决定起到了很大的作用。

我认为媒体中传播的各种阴谋论绝对是胡说八道。(R)

政客和其他领导只是幕后权力操纵的木偶。

大多数人并没有意识到在多大程度上我们的生活在被秘密策划的阴谋所决定。

并没有什么好的理由去不相信政府、情报机构和媒体。(R)

国际情报机构在我们日常生活中起到的作用比我们想象的要大。

秘密组织可以操纵人们的心理，所以人们并没有意识到自己的生活正在被他人控制。

存在一些特定的政治圈，内部有一些非常有影响力的秘密议程。

大部分人不明白我们的生活有多少是被秘密策划的阴谋所决定的。

**Intergroup Conspiracy Theories:**

***English Version***

The secret agency of China has been trying to influence political decision-making in America.

Companies in China are trying to take over America's economy.

The Chinese government is secretly conspiring to harm America.

Chinese secret agencies are trying to use genetically modified organisms as a bio-terrorism weapon to kill Americans.

Chinese secret agencies often try to change the people who are in power in America.

The Chinese government is attempting to interfere with the American government and control the U.S.

The Chinese government is trying to split America from its allies.

***Chinese Version***

美国的秘密机构一直试图影响中国的政治决策。

美国的公司试图控制中国的经济。

美国政府暗中阴谋伤害中国。

美国秘密机构试图利用转基因食品作为杀死中国人的生物恐怖主义武器。

美国的秘密机构正试图改变在中国掌权的人。

美国政府试图干涉中国政府并控制中国。

美国政府正试图将中国从盟国分裂出去。

**Power Distance (Brockner et al., 2001):**

***English Version***

There would be established ranks in society with everyone occupying their rightful place regardless of whether that place is high or low in the ranking.

Even if employees may feel to deserve a salary increase, it would be disrespectful to ask their manager for it.

People are better off not questioning the decisions of those in authority.

Communications with superiors should always be done using formally established procedures.

***Chinese Version***

每个人都应该在社会等级中找到自己的位置，无论这个位置是高还是低。

即使一个员工觉得他应该加薪，但直接向经理要是不好的。

人们最好不要质疑当权者的决定。

应始终按照正式建立的程序与上级沟通。

**Vertical collectivism (Singelis et al., 1995):**

***English Version***

I would sacrifice an activity that I enjoy very much if my family did not approve of it.

I would do what would please my family, even if I detested that activity.

Before taking a major trip, I consult with most members of my family and many friends.

I usually sacrifice my self-interest for the benefit of my group.

Children should be taught to place duty before pleasure.

I hate to disagree with others in my group.

We should keep our aging parents with us at home.

Children should feel honored if their parents receive a distinguished award.

***Chinese Version***

如果家人不赞成，即使我非常喜欢的活动我也不会做。

我会为我的家人去做一些即使我厌恶活动。

在旅行之前，我会询问大多数家人和很多朋友。

为了我团队的利益，我通常牺牲自己的利益。

应教导儿童履行职责比享乐重要。

我讨厌和我小组中的其他人有不同意见。

我们应该把我们年迈的父母留在身边。

如果父母获得杰出奖励，孩子应该感到荣幸。

**Perceived Outgroup Threat:**

***English Version***

China poses a threat to the national interests of the United States.

If China has its way, people in the US will be much worse off.

If China gains economically, the US loses.

***Chinese Version***

美国对中国的国家利益构成威胁。

如果美国人为所欲为的话，那么中国人的情况会更糟。

如果美国经济增长，中国就会损失。

**Collective Narcissism (Federico & de Zavala, 2018):**

***English Version***

If the United States had a major say in the world, the world would be a much better place.

The United States deserves special treatment.

It really makes me angry when others criticize the United States.

Not many people seem to fully understand the importance of the United States.

I will never be satisfied until the United States gets the recognition it deserves.

***Chinese Version***

如果中国在世界上拥有重要的发言权，世界将会变得更加美好。

中国值得特别对待。

当别人批评中国时，我会感到生气。

似乎没有多少人完全理解中国的重要性。

在中国得到应有的认可之前，我永远不会满意。

**Measures Not Analyzed in the Manuscript:**

**Horizontal individualism (Singelis et al., 1995):**

***English Version***

I often do "my own thing".

One should live one's life independently of others.

I like my privacy.

I prefer to be direct and forthright when discussing with people.

I am a unique individual.

What happens to me is my own doing.

When I succeed, it is usually because of my abilities.

I enjoy being unique and different from others in many ways.

***Chinese Version***

我通常自己处理自己的事情。

一个人应该能够独立于别人生活。

我喜欢一个人呆着。

在与他人交谈时，我喜欢直接明确地说出自己的想法。

我是一个独特的人。

我认为，无论发生什么事都是我自己的原因所致。

我的成就通常是凭借自己的能力取得的。

我喜欢在很多方面与众不同。

**Vertical individualism (Singelis et al., 1995):**

***English Version***

It annoys me when other people perform better than I do.

Competition is the law of nature.

When another person does better than I do, I get tense and aroused.

Without competition, it is not possible to have a good society.

Winning is everything.

It is important that I do my job better than others.

I enjoy working in situations involving competition with others.

Some people emphasize winning; I'm not one of them. (R)

***Chinese Version***

当别人表现得比我好时，我会感到恼火。

竞争是自然界的法则。

当别人做得比我好时，我会感到紧张和不安。

没有竞争则无法造就一个良好的社会。

赢，代表一切。

把我自己的工作做得比别人好很重要。

我喜欢在有竞争的环境下学习或工作。

我不是只看重输赢的那类人。(R)

**Horizontal collectivism (Singelis et al., 1995):**

***English Version***

The well-being of my co-workers is important to me.

If a co-worker gets a prize, I would feel proud.

If a relative were in financial difficulty, I would help within my means.

It is important to maintain harmony within my group.

I like sharing little things with my neighbors.

I feel good when I cooperate with others.

My happiness depends very much on the happiness of those around me.

To me, pleasure is spending time with others.

***Chinese Version***

我同事的幸福对我很重要。

如果一个同事获奖，我会感到自豪。

如果有亲戚遇到经济困难，我会尽力帮助。

保持自己团队内部的和谐非常重要。

我喜欢与邻居分享琐事。

当与他人合作时，我感觉良好。

我的幸福在很大程度上取决于我周围的人的幸福。

对我来说，快乐是与他人共度时光。

**Intolerance of Uncertainty (Carleton et al., 2007):**

***English Version***

Unforeseen events upset me greatly.

It frustrates me not having all the information I need.

One should always look ahead so as to avoid surprises.

A small, unforeseen event can spoil everything, even with the best of planning.

I always want to know what the future has in store for me.

I can’t stand being taken by surprise.

I should be able to organize everything in advance.

Uncertainty keeps me from living a full life.

When it’s time to act, uncertainty paralyses me.

When I am uncertain I can’t function very well.

The smallest doubt can stop me from acting.

I must get away from all uncertain situations.

***Chinese Version***

不可预见的事件让我心烦意乱。

没有所需的所有信息让我感到沮丧。

人们应该始终向前看，以避免意外。

即使是最好的规划，一个小的，无法预料的事件也会破坏一切。

我一直想知道我的未来是怎样的。

我不能忍受意料之外的事。

我应该能够提前规划好一切。

不确定性使我无法过上充实的生活。

当要采取行动的时候，不确定性使我动弹不得。

当我不确定时，我不能很好地履行职责。

即使最小的怀疑都可以阻止我采取行动。

我必须远离所有不确定的情况。

**National Identification (Pehrson, Vignoles, & Brown, 2009; Mashuri & Zaduqisti, 2013):**

***English Version***

I feel close to America.

I feel proud of being a(n) American.

The fact that I am a(n) American is an important part of my identity.

I feel connected and attached to other Americans.

**Chinese Version**

我觉得与中国很亲近。

作为中国人，我感到自豪。

中国人是我身份认同的重要组成部分。

我觉得与其他中国人有紧密的联系。

Cultural sample

Power distance values

Collective narcissism

Outgroup threat

Intergroup conspiracy beliefs

.68

.85

.19

.47

.32

.16

.71

**Figure S1.** Parsimonious structural equation model in Study 1 (completely standardized solution). Cultural samples were US (0) and China (1). All paths included gender, age, and education as control variables. All regression coefficients are significant (*p*s < .001). CFI = .920; RMSEA = .077, CI_90%_[.071; .083]; SRMR = .051; χ^2^(209, *N* = 482) = 804.52, *p* < .001.

Cultural sample

Power distance values

Collective narcissism

Outgroup threat

Intergroup conspiracy beliefs

.53

.87

.17

.53

.40

.40

.46

**Figure S2.** Parsimonious structural equation model in Study 2 (completely standardized solution). Cultural samples were US (0) and China (1). All paths included gender, age, and education as control variables. All regression coefficients are significant (*p*s < .001). CFI = .909; RMSEA = .082, CI_90%_[.077; .087]; SRMR = .047; χ^2^(209; *N* = 610) = 1062.19, *p* < .001.
